# Simulating the Kibble-Zurek mechanism of the Ising model with a superconducting qubit system

**DOI:** 10.1038/srep22667

**Published:** 2016-03-08

**Authors:** Ming Gong, Xueda Wen, Guozhu Sun, Dan-Wei Zhang, Dong Lan, Yu Zhou, Yunyi Fan, Yuhao Liu, Xinsheng Tan, Haifeng Yu, Yang Yu, Shi-Liang Zhu, Siyuan Han, Peiheng Wu

**Affiliations:** 1 National Laboratory of Solid State Microstructures, School of Physics, Nanjing University, Nanjing 210093, China; 2 Department of Physics and Astronomy, University of Kansas, Lawrence, KS 66045, USA; 3 Department of Physics, University of Illinois at Urbana-Champaign, Urbana, IL 61801, USA; 4 Research Institute of Superconductor Electronics, School of Electronic Science and Engineering, Nanjing University, Nanjing 210093, China; 5 Synergetic Innovation Center of Quantum Information and Quantum Physics, University of Science and Technology of China, Hefei, Anhui 230026, China; 6 Guangdong Provincial Key Laboratory of Quantum Engineering and Quantum Materials, SPTE, South China Normal University, Guangzhou 510006, China

## Abstract

The Kibble-Zurek mechanism (KZM) predicts the density of topological defects produced in the dynamical processes of phase transitions in systems ranging from cosmology to condensed matter and quantum materials. The similarity between KZM and the Landau-Zener transition (LZT), which is a standard tool to describe the dynamics of some non-equilibrium physics in contemporary physics, is being extensively exploited. Here we demonstrate the equivalence between KZM in the Ising model and LZT in a superconducting qubit system. We develop a time-resolved approach to study quantum dynamics of LZT with nano-second resolution. By using this technique, we simulate the key features of KZM in the Ising model with LZT, e.g., the boundary between the adiabatic and impulse regions, the freeze-out phenomenon in the impulse region, especially, the scaling law of the excited state population as the square root of the quenching speed. Our results provide the experimental evidence of the close connection between KZM and LZT, two textbook paradigms to study the dynamics of the non-equilibrium phenomena.

Non-equilibrium phenomena at avoided level crossings play an essential role in many dynamical processes throughout physics and chemistry. A transition between energy levels at the avoided crossing is known as the Landau-Zener transition (LZT)^1^,^2^, which has served over decades as a textbook paradigm of quantum dynamics. LZT has recently been extensively studied^3^ both theoretically and experimentally in, e.g., superconducting qubits^4^,^5^,^6^,^7^, spin-transistor^8^, and optical lattices^9^,^10^,^11^,^12^. On the other hand, quantum phase transition may also relate to avoided level crossings and it plays an important role in nature. Recently, an elegant theoretical framework for understanding the dynamics of phase transition is provided by the Kibble-Zurek mechanism (KZM)^13^,^14^,^15^,^16^. When the parameters of a quantum system that drive the quantum phase transition are varied in time causing the system to traverse the critical point, KZM predicts that the density of the defects produced in the process follows a power law that scales with the square root of the speed at which the critical point is traversed. Due to its ubiquitous nature, this theory finds applications in a wide variety of systems ranging from cosmology to condensed matter and quantum materials^17^,^18^,^19^,^20^.

The correspondence between LZT and KZM was first pointed out by Damski^21^,^22^. It was shown that the dynamics of LZT can be intuitively described in terms of KZM of the topological defect production in nonequilibrium quantum phase transition. A widely used picture to model the dynamical process of LZT is the adiabatic-impulse approximation (AIA), which was originally developed in KZM theory. The entire dynamical process can be divided into three regions: the adiabatic, impulse, and adiabatic regions, as shown in Fig. 1a. The three regions are separated by two boundaries 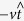 and 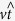, where *v* is the quench rate and 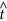 is referred to as the freeze out time. Based on AIA, the dynamics of topological defect production in non-equilibrium phase transitions can be simulated with LZT which was experimentally demonstrated using an optical interferometer^23^. However, some key features of the correspondence between LZT and KZM, such as the freeze out time 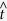 and the adiabatic-impulse-adiabatic regions, have not been investigated experimentally. Most importantly, by studying the dynamical quantum phase transition in a quantum Ising chain, it is found that the average density of defects scales as the square root of the quenching speed^24^,^25^. This universal scaling law of defect formulation as a function of quench speed, which lies at the heart of KZM, lacks adequate experimental evidence in LZT.

In this paper, we use LZT in superconducting qubits to simulate KZM of the Ising model. We develop a time-resolved method to directly investigate the quantum dynamics of LZT in the superconducting qubit. Using state tomography, we measure the time evolution of the population *P*_+_ of the instantaneous positive energy eigenstate [see Fig. 1a] for the entire LZT process. We find that *P*_+_ exhibits a rapid change near the center of the avoided crossing and varies gradually outside this region, revealing the existence of the adiabatic and impulse regions. Moreover, the freeze-out behavior predicted by KZM has also been observed, and the boundary between the adiabatic and impulse region predicted by AIA is confirmed. We observe that the experimental simulated KZM of Ising model displays the theoretically predicted Kibble-Zurek scaling law. Therefore, our result demonstrate the close connection between KZM and LZT, in particular, the presence of Kibble-Zurek scaling behavior in LZT.

## Results

### The equivalence between KZM and LZT

The Ising model is regarded as one of the two prototypical models to understand quantum phase transitions^26^. After rescaling all the quantities to the dimensionless variables, we obtain the Ising model Hamiltonian


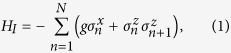


with the periodic boundary condition, where 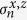 are the Pauli-matrices operators. Here *N* is the total number of spins and *g* is a dimensionless constant driving the phase transition. The ground state of *H*_*I*_ is a paramagnet for 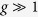 and a ferromagnet for 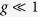, and *g* = 1 corresponds to the critical point. To study the dynamics of this model, we assume that the system evolves from time *t*_*i*_ = −∞ to *t*_*f*_ = 0, and takes a linear quench 
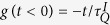
, where  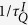 provides a quench rate.

By utilizing Jordan-Wigner transformation and Fourier transform, the Ising model can be simplified as a bunch of decoupled qubits with the Hamiltonian 
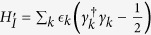
, where the eigenenergy 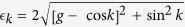 and  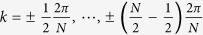 is the pseudomomentum. Here and hereafter we set the lattice constant *a* = 1. The density of defects resulting from the quantum quench has the expression 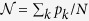, where *p*_*k*_ is the excitation probability corresponding to the pseudomomentum *k*. We consider a special case where the system undergoes slow evolution with 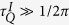. Under this condition, it is safe to assume that only long wavelength modes are excited, i.e., 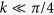, and then 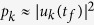, where {*u*_*k*_, *v*_*k*_} are Bogoliubov modes governed by the following matrix equation


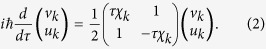


Here 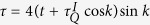 is the normalized time, and 
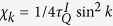
 is the sweeping velocity.

On the other hand, for a quantum two-level system with the diabatic basis, we consider the time-dependent Hamiltonian of a quantum two-level system in the diabatic basis  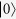 and 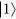



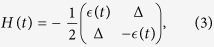


where Δ is the tunneling amplitude and *ϵ*(*t*) is the energy difference between the two diabatic basis. We mainly consider *ϵ*(*t*) = *vt* with *v* being the speed of energy variation. The time-dependent instantaneous energy eigenstates 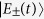 are


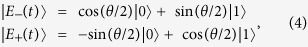


where cos *θ* = *ϵ*(*t*)/Ω(*t*) with 
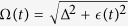
. The instantaneous energy eigenvalues of *H*(*t*) are 
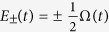
, forming an avoided level crossing at *t* = 0 with a gap Δ. If the system initially in the ground state traverses the avoided crossing, the Landau-Zener theory gives the probability of the qubit occupying the exciting state as *P*_*LZ*_ ≈ exp(−*π*Δ^2^/2 *ħv*). LZT has received tremendous attention since quantum two-level systems, i.e., qubits, are currently considered as the best building blocks of quantum information processors.

With the substitution *v*/Δ^2^ = *χ*_*k*_ and *t*Δ = *τ*, it is found that the dynamics of the Ising model governed by Eq. (2) is the same as LZT physics contained in Eq. (3) up to a normalized tunneling amplitude Δ^25^. Therefore, we can use LZT to simulate KZM of the Ising model.

**Figure 1:**
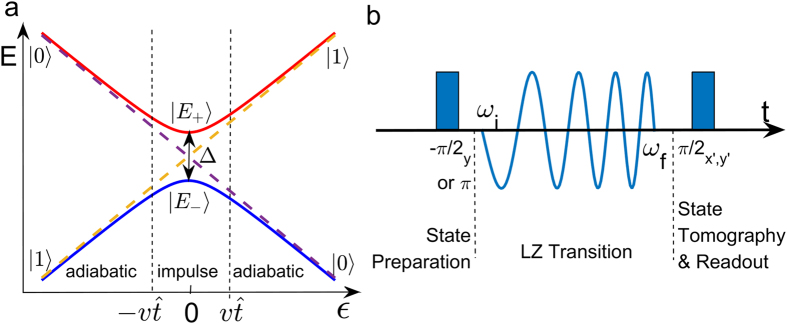
Energy level avoided crossing and experimental procedure. (**a**) A typical energy structure (parameterized by time) of a two-level system. The diabatic states  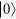, 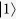, and energy eigenstates 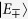 are denoted in the plot. (**b**) A schematic of time profile of the experiment consisting of three parts. During state preparation, a −*π*/2_*y*_ (*π*) pulse is applied to prepare the qubit in 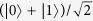 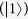. The Landau-Zener transition is realized by chirping the microwave frequency from *ω*_*i*_ to *ω*_*f*_. The final state of the qubit is obtained by state tomography.

A key concept of KZM is AIA. Following the arguments in refs 21,22, we consider two nontrivial schemes to relate LZT with KZM. In scheme **A**, the system starts far away from the avoided crossing, corresponding to *ϵ*_*i*_ → −∞, and ends also far away from the avoided crossing, corresponding to *ϵ*_*f*_ → ∞. The initial state 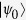 is the ground state of the Hamiltonian at time *t* = −∞. As shown in Fig. 1a, the evolution can be divided into three regions: there are two adiabatic regions 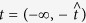 and 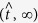, where almost no transition between the instantaneous energy eigenstates 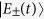 occurs. On the contrary, in the impulse region 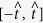 transitions between the states 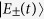 could occur. Quantitatively, the boundaries separating the adiabatic and impulse regions are determined by the freeze-out time 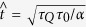, where *α* is a dimensionless parameter, *τ*_*Q*_ = Δ/*v* sets the scale of the quench time and *τ*_0_ = 1/Δ, respectively. The finite density of topological defects 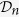 is caused by non-adiabatic evolution in the impulse region  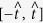, which equals the occupation probability of  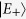. Therefore, the density of topological defects *D*_*n*_ in KZM corresponds to the transition probability *P*_+_ in LZT, i.e., *D*_*n*_ = *P*_+_. In scheme **B**, the system starts from the center of the avoided crossing, i.e., *ϵ*_*i*_ = 0, and evolves to the adiabatic region till far away from the avoided crossing. Similarly, there are two regions, an impulse region 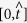 and an adiabatic 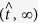, can be defined. For both schemes, we directly measure the time-resolved *P*_+_ of LZT in our experiment and then quantitatively compare the result with the prediction of KZM.

### The time-resolved LZT

We use superconducting qubits to investigate the dynamics of the Ising model. Two samples were studied: a superconducting phase qubit (denoted as *Q*_1_ with *T*_1_ = 113 ns, 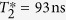)^6^ and a 3D transmon (denoted as *Q*_2_ with *T*_1_ = 2.386 *μ*s, 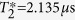, see supplementary material). Here *T*_1_ is the energy relaxation time from state 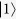 to state  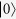, and 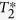 is the decoherence time including contributions from both relaxation and dephasing. Because neither the phase qubit nor the transmon qubit possess an intrinsic avoided level crossing, we use a coherent microwave field to generate an adjustable effective avoided energy level crossing^27^. The position and the tunneling amplitude (Δ) of the avoided crossing are determined by the frequency and amplitude of the microwave field, respectively. With this flexibility and controllability, instead of sweeping the flux bias, we chirp the microwave frequency *ω* while keeping the qubit frequency *ω*_10_ constant to realize LZT (see Methods). The profiles of the control and measurement pulses are illustrated in Fig. 1b.

In our experiment, scheme **A** is a good approximation to LZT from −∞ to +∞ because *ϵ*_*i*_/2*π* = −200 MHz and Δ/2*π* = 20 MHz, resulting  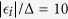. The initial state is prepared in  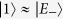 at *ϵ*_*i*_/2*π* = −200 MHz. This is followed immediately by chirping *ϵ* to *ϵ*_*f*_ with a constant speed *v* = (*ϵ*_*f*_ − *ϵ*_*i*_)/*t*_*LZ*_, where *t*_*LZ*_ is the duration of the chirping operation. At the end of *ϵ* chirping, we perform state tomography (see supplementary material) to determine the density matrix of the qubit 
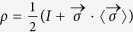
 by measuring the expectation values 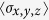, where *σ*_*x*,*y*,*z*_ are the Pauli matrices. We then varied *t*_*LZ*_ from 1 ns to 120 ns, and *ϵ*_*f*_/2*π* from −200 MHz to +400 MHz to obtain a complete set of experimental data. Since the avoided crossing centers at *ϵ* = 0, our experiments effectively cover the dynamical evolution of the system from 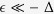 to 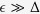.

**Figure 2:**
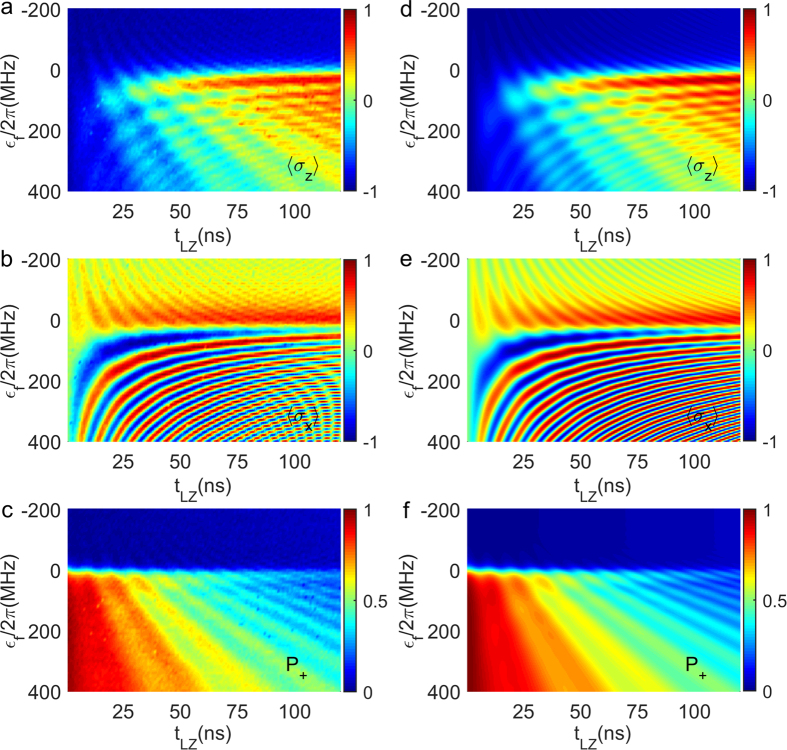
The values of 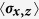 and *P*_+_ as a function of *ϵ*_*f*_/2*π* and *t*_*LZ*_. Here *ϵ*_*i*_/2*π* = −200 MHz and Δ/2*π* = 20 MHz. The range of *ϵ*_*f*_/2*π* is from −200 MHz to 400 MHz. The LZ duration *t*_*LZ*_ is from 1 ns to 120 ns. (**a**–**f**) are the experimental (numerically simulated) results.

By converting the density matrix to the time-dependent basis  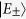, we obtain the


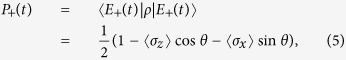


which shows that only 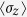 and 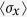 contribute to *P*_+_. The measured 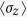, 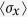 and *P*_+_ for qubit *Q*_2_ are plotted in Fig. 2a–c, respectively. Shown in Fig. 2d–f are the results of numerical simulation obtained by solving the master equations (see Methods), where important system parameters, such as the relaxation and the dephasing times are determined from the pump-decay and the Ramsey fringe measurements. The good agreement between the experimental and the simulated results indicates that all essential aspects of our experiment are well controlled and understood.

### The adiabatic and impulse regions

**Figure 3:**
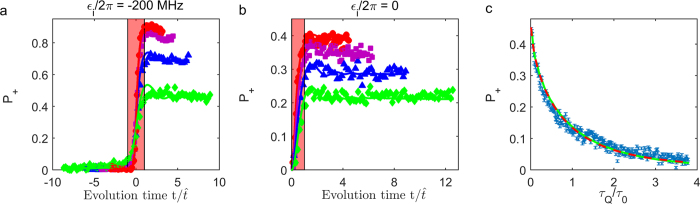
Population *P*_+_ as a function of the normalized time 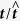 and the comparison of 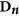 and *P*_+_. (**a**) the evolution starting from t = −∞ with *ϵ*_*i*_/2*π* = −200 MHz and *ϵ*_*f*_/2*π* = 200 MHz. (**b**) the evolution starting from t = 0 with *ϵ*_*i*_/2*π* = 0 and *ϵ*_*f*_/2*π* = 400 MHz. Different LZ durations *t*_*LZ*_ = 10 ns (red circle), 20 ns (magenta square), 40 ns (blue triangle), 80 ns (green diamond), are used to produce different LZT speed. The symbols (solid lines) are experimental (numerical) results. The red translucent (clear) regions mark the impulse (adiabatic) regions, while the boundary locates on 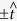. The error bars are smaller than the sizes of the symbols. (**c**) The comparison of topological defects density 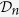 in KZM theory and *P*_+_ in LZT with *ϵ*_*i*_/2*π* = 0. The blue symbols (green solid lines) are the experimental (numerical) results. The red dashed line shows the density 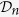 predicted in KZM with *α* = 0.784 as the best fit.

The time resolved quantum dynamics of *P*_+_(*t*) described above can be investigated by measuring  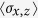 with nano-second time resolution. Shown in Fig. 3a are examples of *P*_+_ for qubit *Q*_2_ as a function of evolution time for various LZT duration time *t*_*LZ*_. In order to compare with AIA, we normalize the evolution time by the freeze-out time 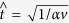. The value of *α* used here is *π*/2, which is the same as that of AIA in this scheme^21^,^22^. In all cases *P*_+_ changes rapidly near the center of the avoided crossing and varies slowly outside the central region. This is the clear experimental evidence supporting the physical picture of AIA. Moreover, the boundaries between the adiabatic and impulse regions are demarcated by 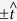 with no fitting parameters, confirming the validity of AIA.

In scheme **B**, we investigate LZT by starting from the center of the avoided crossing (i.e., *ϵ*_*i*_ = 0). The system is initialized in the lower energy eigenstate at *ϵ*_*i*_ = 0 with a proper resonant *π*/2 pulse^28^,^29^,^30^. Then a time sequence similar to that in scheme **A** is applied. Here, *ϵ*_*f*_/2*π* ranges from 0 to 400 MHz. The gap size is fixed at Δ/2*π* = 20 MHz, resulting a maximal *ϵ*_*f*_/Δ = 20. Shown in Fig. 3b are examples of measured *P*_+_ as a function of the evolution time. In this case, *α* = *π*/4 according to refs 21,22. Similar adiabatic and impulse regions are observed with the boundary at 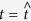, strongly supporting AIA.

### The freeze-out phenomenon

**Figure 4:**
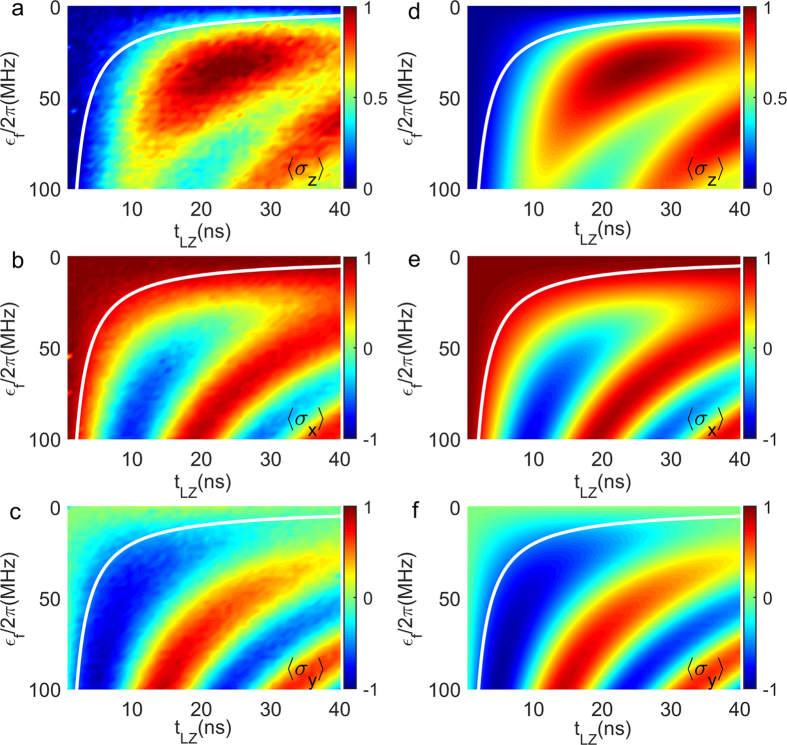
State freeze-out phenomena. (**a**–**f**) are the experimental observation (numerical simulation) of the state freeze-out phenomena of the expectation values 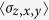 in LZT with *ϵ*_*i*_/2*π* = 0. The white solid line marks the freeze-out time 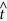 in KZM with *α* = *π*/4.

Another interesting problem is whether one can observe directly the predicted state freeze-out phenomenon in the impulse region 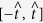. According to KZM, although *P*_±_ of the time-dependent basis states 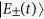 change rapidly in the impulse region, the probability amplitudes (thus 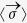) in the time-independent basis 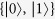 should be frozen out. To see whether this is indeed the case, we plot the measured 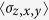 of the qubit *Q*_2_ in Fig. 4a–c (see Methods). The line represents the freeze-out time 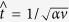 is also shown in the plot. Here we use the theoretical KZM value *α* = *π*/4 because the total LZT duration is shorter than 40 ns and the effect of decoherence may not have significant effects on the result. It can be seen that 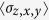 change slowly in the impulse region, indicating that the state of the qubit is nearly frozen. In order to compare with the experimental data, we present the numerically simulated results by solving master equations without adjustable parameters for the evolution of LZT in Fig. 4d–f. The good agreement between the simulation and the experimental results supports the observation of the state freeze-out phenomenon and confirms the validity of AIA.

One of the key features of the correspondence between KZM and LZT is 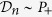. In Fig. 3c we plot *P*_+_ as a function of *t*_*LZ*_ for *ϵ*_*f*_/2*π* = 200 MHz thus 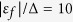. In order to compare with the theory, *t*_*LZ*_ is expressed in terms of *τ*_*Q*_/*τ*_0_. It is found that *P*_+_ follows quite well with the behavior of the topological defects density 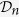 predicted in refs 21,22. Here, *α* = 0.784 is obtained from the best fit which is within 0.2% of KZM predicted value *π*/4. The excellent agreement between the experimental results of LZT and the theory of KZM provides strong support to the conjecture that the dynamics of the Landau-Zener model can be accurately described in terms of the Kibble-Zurek theory of the topological defect production in nonequilibrium phase transitions and vice versa.

### The scaling law

**Figure 5:**
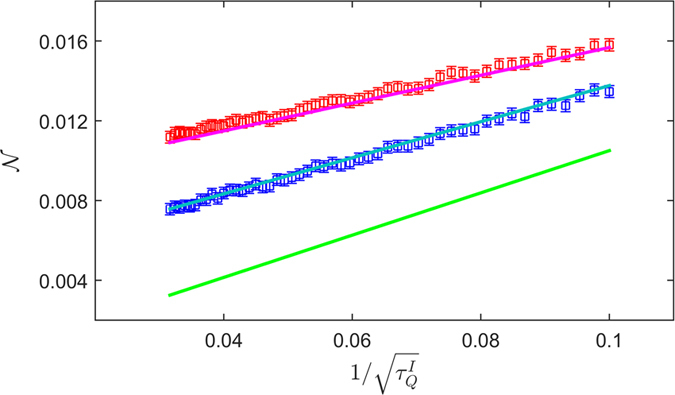
The scaling behavior of 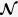 as a function of 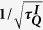. The red (blue) squares represent the experimental data measured in *Q*_1_ (*Q*_2_). The magenta, blue solid lines are the numerical simulation of the master equation with the decoherence of the phase qubit and 3D transmon, respectively. The green solid lines are the simulated results with infinite *T*_1_ and 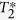.

We now address the simulation of the scaling law predicted by KZM for the Ising model. By choosing small quenching rates  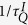, all the relevant physics described by Eq. (2) happens in the long wavelength limit 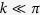. In experiments, we choose a cutoff *k*_*c*_/*π* and thus *v*_*c*_/Δ^2^ = *χ*_*kc*_ to ensure that LZT probability can be neglected for 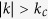. For each 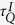, we choose *N*_*k*_ different quasimomentum *k* equally distributed in 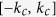, and measure the corresponding excitation probability 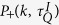. Then the average density of defects can be expressed as


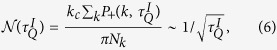


where the last equation is given by KZM theory. Stimulated by this prediction, we plot experimentally measured 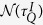 vs. 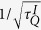 for the qubits *Q*_1_ (red squares) and *Q*_2_ (blue squares) in Fig. 5, where *N*_*k*_ = 127 for each 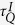, *k*_*c*_/*π* = 0.2 and 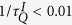. A striking feature is that 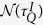 shows a very good linear relation with 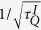. By fitting the line to a general linear function 
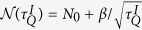
, we obtain the offset *N*_0_ and slope *β* as summarized in Table 1. It is interesting that with the increasing of the decoherence time, the slope increases while the offset decreases. In order to confirm our observation, we did numerical simulations using *T*_1_ and 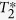 of *Q*_1_, *Q*_2_, and infinite, shown in Fig. 5. Since there is no adjustable parameter, the agreement between the experimental data and numerical simulation results are remarkable. When the decoherence time goes to infinite, the offset tends to zero and the slope 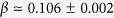, which is very close to the theory predicted value 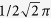
25. Therefore, it is evident that LZT in qubit exhibits same scaling behavior of KZM of the Ising model although decoherence will quantitatively modify the value of some parameters.

## Discussion

In our experiment, the hallmark features of the Ising model predicted by KZM, such as the existence of the adiabatic and impulse regions, the freeze-out phenomenon, and scaling law, were observed. The experimental observations of the first two are in good agreement with the theoretical results because in the experiment the time of evolution is much less than *T*_1_ and 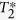 of the qubit *Q*_2_.

We here make several comments on our results of the scaling law. (i) In the absence of decoherence, our numerical results show that the slope 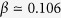, while the theory predicts 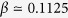. The discrepancy between the numerical and the theoretical results is due to the fact that in the numerical simulation, both *ϵ*_*i*_ and *ϵ*_*f*_ are finite. the LZ transition happens in a finite range, i.e., 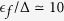. In theory, however, *β* is assuming *ϵ*_*f*_/Δ → ∞. To confirm it, we increased *ϵ*_*f*_/Δ in numerical simulation, and indeed find that *β* approaches 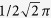 asymptotically. (ii) The offset *N*_0_ in the defect density 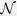 is observed for both qubits in Fig. 5. In addition, the stronger the decoherence, the larger the offset. It was found in previous studies^31^,^32^ that decoherence tends to increase the density of defects. As the decoherence rate increases, more defects are generated in the process of LZ transition. This is confirmed in our experiments, where it is found that the defect density 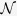 as well as the offset *N*_0_ for qubit *Q*_1_ is greater than that for qubit *Q*_2_. If the coherence time increase further, the offset will gradually approach to zero, as verified by the simulation 3 in Fig. 5. (iii) It is known that the theoretical prediction of ref. 25 is obtained in the continuum limit. So a further question is whether the finite size effect is important in our experiments. If we consider a quantum Ising spin chain with a finite length *N*, there will be energy splitting in the energy spectrum. In this case, if the chirping speed is too slow compared with the energy splitting caused by the finite size, then the prediction of ref. 25 would not be valid. In other words, the finite size effect sets a lower bound on the chirping speed. To be more precise, let us consider the energy splitting near zero energy where 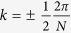. Based on Eq. (2), we require *χ*_*k*=±*π*/*N*_ > 1, corresponding to 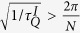. In our experiments, we choose 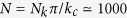, which requires 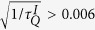, a condition that is very well satisfied in our experiments. Thus the effect of finite size is negligible.

In conclusion, using linear chirps of microwave field and exploring the correspondence between the KZM of topological defects production and LZT, we simulated the KZM of the Ising model with a superconducting qubit system. All important predictions of KZM for the Ising model, such as the existence of adiabatic and impulse regions, the freeze-out phenomenon, and especially the scaling law have been clearly demonstrated. The observed scaling behavior in the presence of decoherence sheds new light on the investigation of the effects of decoherence on KZM of non-equilibrium quantum phase transitions.

## Methods

### Chirp-LZT operation

In order to perform the LZT, we chirp the microwave frequency^29^,^30^ instead of sweeping the flux bias of the qubit. Concretely, with the qubit dc-biased at a fixed flux Φ, we chirp the microwave frequency from *ω*_*i*_ to *ω*_*f*_, corresponding to the change of *ϵ* from *ϵ*_*i*_ to *ϵ*_*f*_. Note that in our experiment, the energy difference is *ϵ*_*i*,*f*_ = *ħ*(*ω*_01_ − *ω*_*i*,*f*_), with a chirping microwave frequency 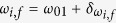. Here we assume *ħ* = 1. If we set the original microwave frequency as *ω*_01_, then the chirped frequency is 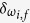. Therefore, the relationship between the chirped microwave frequency 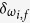 and the diabatic energy difference *ϵ*_*i*,*f*_ is given by 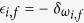.

To chirp the microwave frequency, we apply modulation signals from a Tektronix AWG70002 to the IF (intermediate frequency) ports of a IQ mixer. Considering the original microwave waveform as *A*_*r*_ sin *ω*_0_*t*, the modulation signals applied to the I and Q ports of the IQ mixer as cos *δ*_*ω*_*t* and sin *δ*_*ω*_*t*, respectively. In this way, the modulated microwave at the output port of the mixer is 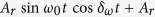 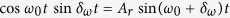, and the microwave frequency is chirped by tuning *δ*_*ω*_. From calibration, we find that the power of *ω*_0_ tone is at least 50 dB lower than that of *ω*_0_ + *δ*_*ω*_, indicating the negligible effect of *ω*_0_ on the qubit in the chirp operation.

The Chirp-LZT method provides us several advantages in performing LZT. First of all, all parameters of the qubit, such as *T*_1_, 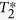 and the coupling strength between the qubit and the external driven filed, are fixed during the measurements. Second, the end points of diabatical energy sweep *ϵ*_*i*_ and *ϵ*_*f*_ would not be limited by the avoided level crossings resulting from the coupling between the qubit and microscopic two level systems (TLSs) usually presented in superconducting qubits because the microwave couples much weakly to TLSs than to the qubit. Third, it is easy to control the chirping velocity and the tunnel splitting Δ by controlling the frequency and power of the microwave. In addition, such method provides a useful tool for systems without natural avoided crossings in their energy diagram, such as transmons, to perform LZT and other similar experiments.

### Solution of the master equation

The numerical results are obtained by solving master equations. The quantum dynamics of the superconducting qubits is described by the master equations of the time evolution of the density matrix *ρ* including the effects of dissipation: 
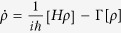
, where *H* is the Hamiltonian of the system described by Eq. (3). The second term of this equation, Γ[*ρ*], describes the effects of decoherence on the evolution phenomenologically. Setting *ħ* = 1, the master equation can be rewritten as


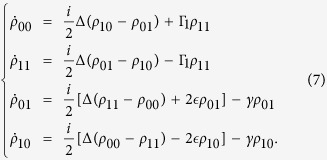


Here Γ_1_ ≡ 1/*T*_1_ is the energy relaxation rate, and 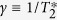 is the decoherence rate. The relationship between the density matrix and the qubit state expectation values are given by


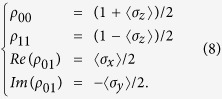


The numerical simulations in Figs 2, 3, 4 are straightforwardly obtained by solving Eq. (7). The simulations (solid lines) in Fig. 5 are obtained by mapping *P*_+_ as defined in Eq. (5) to the defect density 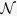. By using *v*/Δ^2^ = *χ*_*k*_ for each chirping velocity *v*, we can find the corresponding momentum *k* with 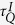 fixed. In this way, we obtain 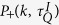 for different momentum *k*. Then 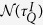 is computed from *P*_+_ using Eq. (6).

## Additional Information

**How to cite this article**: Gong, M. *et al*. Simulating the Kibble-Zurek mechanism of the Ising model with a superconducting qubit system. *Sci. Rep.* **6**, 22667; doi: 10.1038/srep22667 (2016).

## Supplementary Material

Supplementary Information

## Figures and Tables

**Table 1:** The offset *N*_0_ and slope *β* extracted from the experimental (*Q*_1_, *Q*_2_) and simulated results (Simu. 1, Simu. 2, Simu. 3).

Samples	*T*_1_	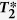	*β*	*N*_0_
*Q*_1_	113 ns	93 ns	0.068 ± 0.002	0.0091 ± 0.0001
*Q*_2_	2.386 *μ*s	2.135 *μ*s	0.088 ± 0.002	0.0048 ± 0.0001
Simu. 1	113 ns	93 ns	0.070 ± 0.001	0.0087 ± 0.0001
Simu. 2	2.386 *μ*s	2.135 *μ*s	0.090 ± 0.002	0.0048 ± 0.0001
Simu. 3	∞	∞	0.106 ± 0.002	−0.0001 ± 0.0001
