# Supplementary material for: Simulating the Kibble-Zurek mechanism of the Ising model with a superconducting qubit system
Source: Sci Rep. 2016 Mar 8;6:22667. doi: 10.1038/srep22667 (PMC4782105; doi:10.1038/srep22667)
Supplement: Supplementary Information [file srep22667-s1.pdf]

Supplementary material for:
Simulating the Kibble-Zurek mechanism of the Ising model with a superconducting qubit system

Ming Gong[1,2,*], Xueda Wen[3,*], Guozhu Sun[4,5,*], Danwei Zhang[6], Dong Lan[1], Yu Zhou[4], Yunyi Fan[4], Yuhao Liu[1], Xinsheng Tan[1], Haifeng Yu[1,5], Yang Yu[1,5,†], Shiliang Zhu[1,5,‡], Siyuan Han[2,§], Peiheng Wu[4,5]

[1]*National Laboratory of Solid State Microstructures, School of Physics, Nanjing University, Nanjing 210093, China*

[2]*Department of Physics and Astronomy, University of Kansas, Lawrence, KS 66045, USA*

[3]*Department of Physics, University of Illinois at Urbana-Champaign, Urbana, IL 61801, USA*

[4]*Research Institute of Superconductor Electronics, School of Electronic Science and Engineering, Nanjing University, Nanjing 210093, China*

[5]*Synergetic Innovation Center of Quantum Information and Quantum Physics, University of Science and Technology of China, Hefei, Anhui 230026, China*

[6]*Guangdong Provincial Key Laboratory of Quantum Engineering and Quantum Materials, SPTE, South China Normal University, Guangzhou 510006, China*

1 3D Transmon Qubit Sample

In our experiment, the transmon, as shown in Fig. S1a, is patterned with a 30 KeV e-beam lithography, followed by double-angle shadow evaporation of aluminum (thicknesses of layers are 30 and 80 nm, respectively) and lift-off procedures to form the final structure on a high-resistivity silicon substrate. An optimal oxidation condition is chosen so as to get the proper critical current of Josephson junction, which results in the Josephson energy $E_J/h = 16.7 \pm 0.1$ GHz. From the spectroscopy measurement, we independently determined the charge energy $E_C/h = 274 \pm 2$ MHz. The transmon qubit sample is centered in a 3D rectangular aluminum (Al 6061-T6 alloy) cavity, as shown in Fig. S1b, with the bare resonant frequency as 9.0131 GHz. The sample is mounted in a dilution refrigerator and measured at about 20 mK.

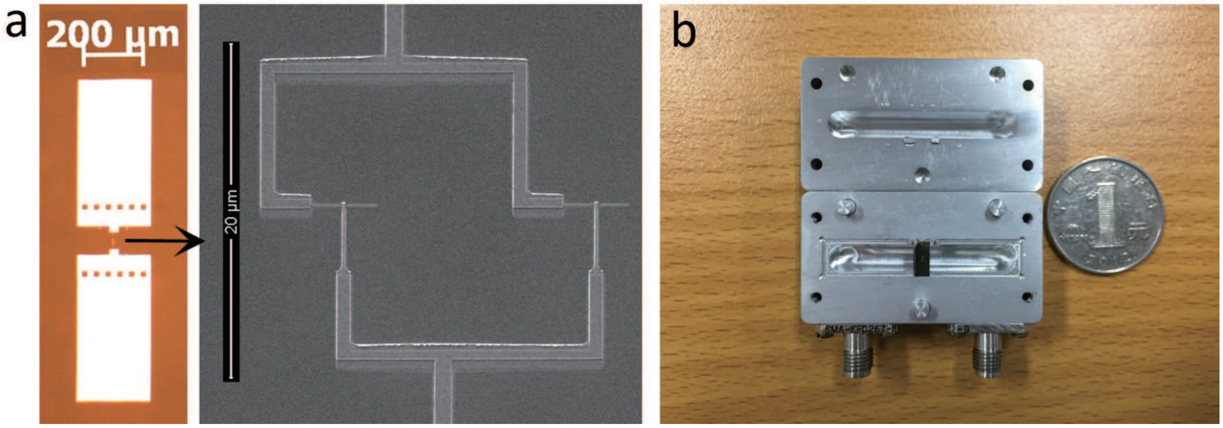

Figure S1 Pictures of the transmon and the cavity. (a) Optical micrograph of 3D transmon qubit. **(b)** The 3D rectangular aluminum cavity with a sample centered in it.

2 Readout and State Tomography Measurement

For the 3D transmon qubit sample, a standard "bright state"readout with heterodyne technique [1] is used to obtain the states of the qubit. The input signal is heavily attenuated and the output signal is amplified more than 20 dB with microwave amplifier [2] to achieve high SNR. We calibrated the measurement setup and find that the readout fidelity is 74%.

Different from the state tomography measurement with a fixed microwave frequency, the dynamic phase θ_f of the Bloch Sphere basis generated by the varying rotation frame should be taken into account in the process of chirping. With the frequency difference between the microwave and the qubit resonant frequency determined as $\delta_\omega(t)$, the dynamic phase is given by $\theta_f(t) = \int_0^t \delta_\omega(\tau)\mathrm{d}\tau$. Then, the new X' and Y' axes to apply a resonant $\pi/2$ pulse for rotating the qubit about is given by $\vec{X'} = cos\theta_f\vec{X} + sin\theta_f\vec{Y}$ and $\vec{Y'} = -sin\theta_f\vec{X} + cos\theta_f\vec{Y}$, where $\vec{X}$ and $\vec{Y}$ are the Bloch Sphere bases with a fixed microwave frequency. After the $\pi/2$ pulse rotation, a standard readout pulse is applied and the qubit state tomography is achieved.

1. Reed, M. D. *et al.* High-fidelity readout in circuit quantum electrodynamics using the jaynes-cummings nonlinearity. *Phys. Rev. Lett.* **105,** 173601 (2010).

2. Rigetti, C. *et al.* Superconducting qubit in a waveguide cavity with a coherence time approaching 0.1 ms. *Phys. Rev. B* **86,** 100506(R) (2012).
